# Focus on the NLRP3 inflammasome: its role in the pathogenesis and therapeutic potential of inflammation-driven osteoarthritis

**DOI:** 10.3389/fimmu.2026.1938807

**Published:** 2026-09-24

**Authors:** Fengyuan Li, Min Tan, Rongjie Zhai, Yuhong Ouyang, Haili Shen

**Affiliations:** 1 The Second Clinical Medical College of Lanzhou University, Lanzhou, China; 2 Department of Rheumatology, Lanzhou University Second Hospital, Lanzhou, China

**Keywords:** inflammation, innate immunity, NLRP3 inflammasome, osteoarthritis, precision medicine, pyroptosis

## Abstract

Osteoarthritis (OA) is a highly heterogeneous whole-joint disease. Current treatment strategies, which primarily focus on symptomatic pain relief, are insufficient to address the therapeutic needs of patients with distinct clinical phenotypes. Accumulating evidence has demonstrated that the empirical use of conventional synthetic disease-modifying antirheumatic drugs (csDMARDs) has shown limited efficacy in OA cohorts selected on the basis of inflammatory features, underscoring the need to more precisely define inflammation-driven OA and elucidate its underlying pathogenic mechanisms. Persistent local inflammation is increasingly recognized as a prominent feature of inflammation-driven OA. Rather than being merely secondary to mechanical injury or degenerative changes, this inflammatory state is associated with sustained release of damage-associated molecular patterns (DAMPs), aberrant activation of innate immunity, and amplification of inflammatory signaling. However, no consensus criteria have been established to define inflammation-driven OA. The NOD-like receptor family pyrin domain-containing 3 (NLRP3) inflammasome integrates diverse danger signals, including extracellular ATP, crystal deposition, ionic fluxes and mitochondrial oxidative stress. Its activation promotes caspase-1 activation, the maturation and release of inflammatory cytokines, and gasdermin D (GSDMD)-mediated pyroptosis. Through cell type-specific responses and intercellular crosstalk within the OA joint microenvironment, aberrant NLRP3 inflammasome activation may sustain local inflammatory amplification, contribute to OA progression, and exacerbate joint tissue damage. In this review, we discuss the basis for stratifying inflammation-driven OA and the challenges associated with its treatment, delineate the potential pathological role of the NLRP3 inflammasome, and explore its potential as both a candidate mechanism-based biomarker and a therapeutic target. We propose that NLRP3 inflammasome activation may provide a mechanistically informative dimension for OA stratification, thereby offering a conceptual framework for moving beyond conventional symptom-oriented management toward mechanism-informed precision intervention.

## 1 Introduction

Osteoarthritis (OA) is a common musculoskeletal disorder that causes chronic pain, restricted joint mobility, and functional impairment, while its clinical management remains marked by substantial unmet needs (1). Current treatment strategies primarily focus on pain relief, improvement of joint function, and management of modifiable risk factors, such as body weight. Joint replacement is generally considered for patients with advanced disease who remain symptomatic despite non-surgical treatment (2). Although the development of disease-modifying osteoarthritis drugs (DMOADs) has progressed in recent years, no approved DMOADs have yet been shown to reliably halt or reverse OA progression (3). Accumulating evidence indicates that OA should not be regarded as a disease with a uniform pathological basis. Across individuals, the relative contributions of abnormal mechanical loading, metabolic dysregulation, synovial inflammation, disturbances in cartilage and bone homeostasis, and pain-related mechanisms vary during disease progression. This heterogeneity is increasingly recognized as an important contributor to variable treatment responses (4, 5). Accordingly, OA research is shifting from the search for universally effective therapies toward mechanism-informed phenotypes and endotypes, with the aim of identifying patient subgroups that are more likely to benefit from specific interventions (5, 6). Inflammation-driven OA has emerged as an important focus of OA phenotype characterization and precision intervention research because of its complex pathophysiology and the absence of unified criteria (7, 8). Within the OA joint microenvironment, the persistent release of damage-associated molecular patterns (DAMPs) can engage pattern-recognition receptors (PRRs) on innate immune cells, including macrophages, thereby promoting inflammatory mediator release, sterile inflammatory responses, and tissue catabolism (9, 10). The NOD-like receptor family pyrin domain-containing 3 (NLRP3) inflammasome functions as an important innate immune signaling platform that integrates diverse danger-associated signals. Subsequent activation of the NLRP3 inflammasome promotes caspase-1-dependent maturation and release of IL-1β and IL-18 and induces GSDMD-mediated pyroptosis, thereby contributing to the initiation and progression of inflammation in OA (11, 12). Although the pathogenic role of the NLRP3 inflammasome in OA has been extensively investigated, it remains unclear whether measures of NLRP3 inflammasome activation can be developed and validated as research biomarkers for mechanism-informed OA stratification or used to predict responses to NLRP3-targeted therapy (7, 8). In the context of OA heterogeneity and the limitations of current anti-inflammatory therapies, we summarize the rationale for stratifying inflammation-driven OA, with particular emphasis on its underlying innate immune mechanisms. We focus on the modes of NLRP3 inflammasome activation within the OA joint microenvironment, its cell type-specific roles, and its contribution to inflammatory amplification. We further assess NLRP3-related activity as a candidate research biomarker for mechanism-informed OA stratification and examine the NLRP3 pathway as a potential therapeutic target, while considering the current limitations of clinical translation.

## 2 Overview of osteoarthritis: a heterogeneous whole-joint disease

OA is a joint disease characterized by progressive degeneration and destruction of articular cartilage, together with osteophyte formation (13). In recent years, the global prevalence of OA has continued to increase, driven by multiple factors including population aging, obesity, and joint injury (14, 15). OA was long regarded as a wear-related or degenerative joint disorder. However, accumulating evidence indicates that OA is a multifactorial chronic joint disease in which immune responses, metabolic dysregulation, inflammatory processes, and mechanical stress collectively contribute to disease initiation and progression (10, 13, 16). Accordingly, OA is no longer considered to be confined to articular cartilage, but rather represents a whole-joint disorder involving cartilage, bone, synovium, ligaments, the joint capsule, and periarticular muscles. Recent studies have proposed that OA may be conceptualized at two interrelated levels: disease and illness. Disease primarily refers to pathological alterations in joint tissues, with imaging-detectable structural damage representing its central feature. In contrast, illness reflects the clinical burden experienced by patients, including pain, stiffness, and functional limitation (17, 18). However, the severity of symptoms does not necessarily correspond to the extent of structural damage detected by imaging, highlighting the marked heterogeneity and complexity of OA. Therefore, conventional treatment strategies focused predominantly on symptom relief may not adequately address the diverse clinical needs of individual patients. Establishing clinically meaningful stratification approaches for OA has thus become an urgent challenge in this field.

## 3 Phenotypes and endotypes of OA: the basis for stratified management

Phenotypes refer to sets of clinically observable or measurable characteristics, including symptoms, signs, and imaging findings. Endotypes are defined by specific cellular states, dysregulated molecular pathways, and tissue- or biofluid-derived biomarkers, and describe the pathobiological mechanisms underlying the corresponding phenotypes (19, 20). Stratifying patients with OA according to phenotypes and endotypes may improve the precision of individualized treatment. However, given the marked heterogeneity of OA, no unified classification framework has yet been established (21–23). Several phenotype-based classification systems have been proposed. One study categorized knee OA into inflammatory, bone and cartilage metabolism, minimal joint disease, chronic pain, metabolic syndrome, and mechanical overload phenotypes (24). The Check-list Osteoarthritis Classification (COACH) project classified OA into load-driven, structure-driven, inflammation-driven, metabolism-driven, systemic factor-driven, and mixed phenotypes (25). Other studies have proposed synovitis-driven inflammatory, metabolic, age-driven, traumatic injury-driven, and subchondral bone-driven phenotypes according to the predominant pathological drivers of disease (23). These classification systems integrate clinical manifestations, structural involvement, and risk factors. Therefore, they may be more appropriately regarded as mechanism-informed phenotypes rather than fully validated endotypes. Biomarker-based unsupervised clustering provides a more direct basis for defining molecular endotypes in OA. In the IMI-APPROACH cohort, knee OA was classified into low tissue turnover, structural damage, and systemic inflammation subgroups based on 16 biochemical biomarkers reflecting tissue metabolism, structural damage, and inflammatory processes. These subgroups were associated with non-progression, structural progression, and persistent or progressive pain, respectively (5). Importantly, OA phenotypes and endotypes do not exhibit a strict one-to-one relationship. Individual patients may present with features of multiple clinical phenotypes and harbor several overlapping pathological mechanisms. As proposed by Hannani et al., distinct OA endotypes may converge on similar phenotypes, whereas a single phenotype may arise from different molecular endotypes (6). This overlap may contribute to the heterogeneous responses of patients to the same therapeutic strategy and further highlights the importance of OA stratification-based management. Among the proposed subgroups, inflammation-driven OA has attracted considerable attention because of detectable synovitis, inflammation-related biomarkers, and its potential amenability to therapeutic intervention. It has therefore emerged as an important focus of precision stratification research in OA (26, 27). In this review, inflammation-driven OA is considered a research-level concept used to describe an OA clinical phenotype in which inflammatory processes may substantially contribute to disease development and progression, rather than a molecular endotype that has been sufficiently validated.

## 4 Current evidence and therapeutic challenges in inflammation-driven OA

Several cohort studies have reported OA phenotypes characterized by inflammatory features, with prevalence estimates ranging from approximately 16% to 60%, suggesting substantial heterogeneity among different study populations and phenotype definitions (28, 29). These phenotypes are generally characterized by excessive expression of inflammatory cytokines and increased imaging evidence of inflammation in the synovium and joint effusion on ultrasound (US) or magnetic resonance imaging (MRI), commonly described as effusion-synovitis (30). A cross-sectional analysis of surgical specimens from 244 patients with end-stage OA undergoing hip or knee arthroplasty further reported that 64.3% of patients exhibited high-grade synovitis. Among them, 55.3% displayed a lympho-myeloid pathotype characterized by the co-infiltration of T and B lymphocytes and myeloid cells, which was significantly associated with higher systemic inflammation indices and C-reactive protein (CRP) levels (31). However, these observations alone do not establish inflammation as the primary driver of disease in these patients. An earlier histopathological study comparing OA synovium with synovium from mechanically induced or trauma-related joint lesions found that inflammatory alterations, including lymphoid follicle formation, diffuse fibrosis, and perivascular fibrosis, were more common in OA synovium. Inflammatory cell infiltration was not clearly associated with locally detected bone or cartilage fragments, or with calcium pyrophosphate crystals. These findings suggest that synovial inflammation in a subset of patients with OA cannot be simply interpreted as a secondary response to debris derived from damaged bone or cartilage. Immunohistochemical analyses of ectopic lymphoid follicle-like structures in knee OA synovium identified CD4^+^T cells, CD8^+^T cells, B cells, and HLA-DR^+^ macrophages. The CD4:CD8 ratio ranged from 1:1 to 2.5:1, differing from the marked CD4^+^T cell enrichment typically observed in rheumatoid arthritis (RA) synovium (32). More recently, the multicenter, randomized, double-blind, placebo-controlled MESKO trial in China showed that low-dose methotrexate (MTX; ≤15 mg/week) did not provide significant benefits over placebo after 52 weeks with respect to pain relief or MRI-assessed synovitis and joint effusion in inflammatory knee OA. Although a trend toward symptom improvement was observed in the subgroup with severe pain, this finding requires further validation because of the limited sample size (33). These findings indicate that low-dose MTX did not demonstrate a clear clinical benefit under the patient selection criteria and treatment regimen applied in the MESKO trial. However, patients in this study were not stratified according to molecular inflammatory endotypes, and MRI-detected effusion-synovitis may not fully capture the underlying molecular inflammatory drivers. Therefore, the specific mechanisms contributing to the limited therapeutic response cannot be determined based on these findings alone. Interleukin-1 (IL-1) plays an important role in OA pathogenesis by promoting cartilage degradation, synovial inflammation, and pain-related signaling. Accordingly, IL-1-targeted strategies have long been regarded as promising therapeutic approaches (34). However, accumulating evidence in recent years has demonstrated that neither IL-1 receptor antagonists nor anti-IL-1β monoclonal antibodies, such as canakinumab, have consistently achieved clinical benefits in previous OA studies (31, 35, 36). These findings suggest that IL-1-targeted therapies have not yet demonstrated reproducible clinical efficacy in OA, and the underlying mechanisms contributing to the limited therapeutic response remain to be fully elucidated. The reasons for the suboptimal therapeutic outcomes are likely multifactorial. On the one hand, the lack of phenotypic stratification in previous OA studies may have resulted in failure to identify patient subsets potentially more responsive to IL-1-targeted interventions. On the other hand, factors including drug exposure regimens, timing of administration, selection of outcome measures, and the intrinsic heterogeneity of OA may have substantially influenced clinical outcomes. Whether mechanism-based patient stratification can improve therapeutic responses in OA requires further validation in prospective clinical trials. Overall, converging evidence from histopathological, imaging, and other studies supports the existence of an inflammation-active subgroup of patients with OA. However, a unified and validated definition for this subgroup has not yet been established, and the limited efficacy of current anti-inflammatory strategies further suggests that its underlying pathological mechanisms remain to be fully elucidated. Taken together, the limited or inconsistent efficacy of current anti-inflammatory and immunomodulatory therapies suggests that descriptive inflammatory phenotypes alone are insufficient to explain the persistence of inflammation in OA, highlighting the need to further elucidate the underlying molecular mechanisms. Independent histological, transcriptomic, and cell-based studies suggest that macrophages and DAMP-mediated innate immune signaling may contribute to persistent synovial inflammation in OA, although the precise mechanisms remain to be clarified.

## 5 Contribution of innate immunity to inflammation-driven OA

Compared with the prominent T/B lymphocyte activation and adaptive immune features observed in RA synovium, studies of human OA synovial tissues have identified macrophages as one of the predominant immune cell populations, with additional involvement of other myeloid cells, suggesting that innate immunity may play an important role in the local inflammatory microenvironment of OA (37–39). In RA, methotrexate (MTX) is a cornerstone therapeutic agent that competitively inhibits dihydrofolate reductase, thereby blocking the conversion of dihydrofolate (FH2) to tetrahydrofolate (FH4), disrupting folate-dependent nucleotide synthesis, and consequently suppressing the proliferation of activated lymphocytes (37, 40, 41). Single-cell sequencing studies have identified clonally expanded CD4+ peripheral helper T cells in RA synovium, supporting the central role of dysregulated adaptive immunity in RA pathogenesis (37). In contrast, macrophages and other innate immune-derived myeloid cells in OA synovial tissues may contribute to synovitis and cartilage damage through the production of inflammatory mediators, chemokines, and tissue-remodeling signals (42, 43). Integrated analyses combining differentially expressed genes, immune-infiltration profiling, and single-cell RNA sequencing (scRNA-seq) identified macrophages, memory B cells, B cells, and mast cells as immune cell populations significantly associated with OA. Least absolute shrinkage and selection operator analysis further identified macrophages as the immune infiltration-associated cell type with the greatest diagnostic value (44). Studies combining scRNA-seq with flow cytometry have further revealed marked heterogeneity among synovial macrophages in OA. Their activation states appear to reflect dynamic adaptation to the joint microenvironment. Macrophages in healthy joints predominantly exhibit M2-like characteristics, whereas OA progression is associated with a shift toward an M1-like pro-inflammatory state. These findings suggest that dysregulated macrophage polarization may represent an important mechanism underlying persistent synovitis (45). At the molecular level, the OA joint microenvironment contains a diverse range of endogenous DAMPs, including cartilage matrix degradation products, such as hyaluronan oligosaccharides and chondroitin sulfate-derived fragments, crystalline materials, mitochondrial reactive oxygen species (mtROS), and high-mobility group box 1 (HMGB1). These DAMPs can be recognized by Toll-like receptor (TLR)2 and TLR4 on synovial macrophages and chondrocytes. Through MyD88-dependent signaling, TLR activation promotes nuclear factor-κB (NF-κB) activation and sustained production of pro-inflammatory cytokines, including IL-1β, tumor necrosis factor-α (TNF-α), and IL-6. These signals subsequently increase the expression of matrix metalloproteinases (MMPs) and other catabolic mediators, thereby aggravating cartilage degeneration and establishing a positive feedback loop between inflammation and tissue injury (46–49). This low-grade inflammatory state associated with innate immune activation suggests that OA pathology cannot be fully explained by wear-related or degenerative joint damage alone and may represent one mechanism contributing to inflammation-driven OA (49). The TLR-NF-κB pathway, a central upstream signaling axis of innate immunity, provides the priming signal for inflammasome-dependent inflammatory responses at the transcriptional level by inducing the expression of pro-inflammatory cytokine precursors, including pro-IL-1β and pro-IL-18 (50, 51). However, the generation of mature IL-1β and IL-18 requires subsequent proteolytic processing (50, 52–54). The NLRP3 inflammasome is a cytosolic multiprotein complex assembled around the pattern-recognition receptor NLRP3. Within the OA joint microenvironment, the NLRP3 inflammasome integrates diverse danger signals, including extracellular ATP, disruption of ion homeostasis, crystal-induced lysosomal damage, and mitochondrial stress, and mediates downstream inflammatory responses through caspase-1 activation (50, 51, 55–57). Therefore, NLRP3 represents a critical molecular node linking DAMP recognition to inflammatory execution. Its activation requires not only transcriptional priming but also subsequent inflammasome assembly and caspase-1 activation, thereby enabling the maturation and release of IL-1β and IL-18 (56, 58).

## 6 The NLRP3 inflammasome in inflammation-driven OA: mechanistic evidence and biomarker potential

### 6.1 Overview of the NLRP3 inflammasome

#### 6.1.1 Structure of the NLRP3 inflammasome

The NLRP3 inflammasome represents a critical regulatory node in OA-associated inflammatory responses (59). It is primarily composed of the sensor protein NLRP3, the adaptor apoptosis-associated speck-like protein containing a CARD (ASC), and the effector protein pro-caspase-1 (60). Upon recognition of activating stimuli, ASC functions as a molecular bridge: its N-terminal pyrin domain (PYD) interacts with NLRP3, whereas its C-terminal caspase recruitment domain (CARD) recruits pro-caspase-1. The close proximity of recruited pro-caspase-1 molecules promotes their autoactivation and cleavage into active caspase-1. Active caspase-1 subsequently cleaves pro-IL-1β and pro-IL-18 into their mature forms, which are released into the extracellular milieu to mediate inflammatory responses (61, 62). In parallel, active caspase-1 cleaves gasdermin D (GSDMD), thereby releasing its pore-forming N-terminal fragment. GSDMD-N oligomerizes within the plasma membrane to form pores, facilitating the release of mature cytokines and inducing pyroptotic cell death. This process contributes to inflammatory tissue injury within the joint microenvironment (63, 64).

#### 6.1.2 Canonical and non-canonical activation pathways

NLRP3 inflammasome activation depends on tightly regulated molecular events and can be broadly classified into canonical and non-canonical pathways according to differences in their upstream triggers and downstream effector molecules (65). These pathways differ substantially in their initiating signals and signaling components.

##### 6.1.2.1 Canonical pathway

The canonical NLRP3 inflammasome pathway is generally considered a two-step process involving a priming signal followed by an activation signal (66). The first signal, also referred to as the priming signal, is induced by pathogen-associated molecular patterns (PAMPs) or DAMPs. These stimuli activate receptors such as TLRs, IL-1 receptors, and tumor necrosis factor receptors (TNFRs), leading to NF-κB activation. NF-κB primarily promotes the transcriptional expression of NLRP3, pro-IL-1β, and, in certain cell types, pro-IL-18 (67). In addition to transcriptional regulation, NF-κB-related signaling may facilitate NLRP3 activation through post-translational modifications, including deubiquitination and phosphorylation (68). Furthermore, Fas-associated death domain protein (FADD) and caspase-8 can participate in this priming process as upstream regulatory molecules, independently of K^+^ efflux or pyroptosis (69).

The second signal, also referred to as the activation signal, can be elicited by structurally diverse stimuli, including extracellular ATP, pore-forming toxins such as nigericin, particulate materials such as silica and monosodium urate crystals, and endogenous amyloid aggregates (70). These stimuli promote NLRP3 inflammasome activation through several interconnected mechanisms. Firstly, K^+^ efflux is one of the most common and essential triggering events (71). Extracellular ATP can activate the P2X7 purinergic receptor, whereas pore-forming toxins can directly disrupt plasma membrane integrity. Both processes decrease intracellular K^+^ concentrations and promote NLRP3 conformational changes and oligomerization through its interaction with NIMA-related kinase 7 (NEK7) (72). Secondly, chloride channels also contribute to NLRP3 activation. During inflammasome activation, chloride intracellular channel (CLIC) proteins, particularly CLIC1 and CLIC4, translocate to the plasma membrane and mediate Cl^−^ efflux. This process acts cooperatively with K^+^ efflux to facilitate NLRP3 inflammasome activation, although Cl^−^ efflux alone is insufficient to activate caspase-1 (73, 74). Thirdly, lysosomal rupture represents a major mechanism through which particulate stimuli activate NLRP3. Following phagocytosis, crystals or particles increase lysosomal membrane permeability and promote the release of cathepsin B (a lysosomal cysteine protease) into the cytosol. Cytosolic cathepsin B can subsequently contribute to inflammasome assembly (75). In addition to cathepsin B release, particle-dependent phagocytosis can induce K^+^ efflux. Multiple studies indicate that lysosomal damage-mediated NLRP3 activation remains dependent on K^+^ loss (75–77). Finally, mitochondrial dysfunction frequently accompanies these processes. Increased mtROS production and the release of oxidized mitochondrial DNA (ox-mtDNA) through the mitochondrial permeability transition pore may promote NLRP3 activation (78). Together, these signals facilitate the assembly of NLRP3, ASC, and pro-caspase-1 into a functional inflammasome complex, leading to caspase-1 activation and the subsequent maturation and release of IL-1β and IL-18 (70, 79, 80) (**Figure 1**).

**Figure 1:**
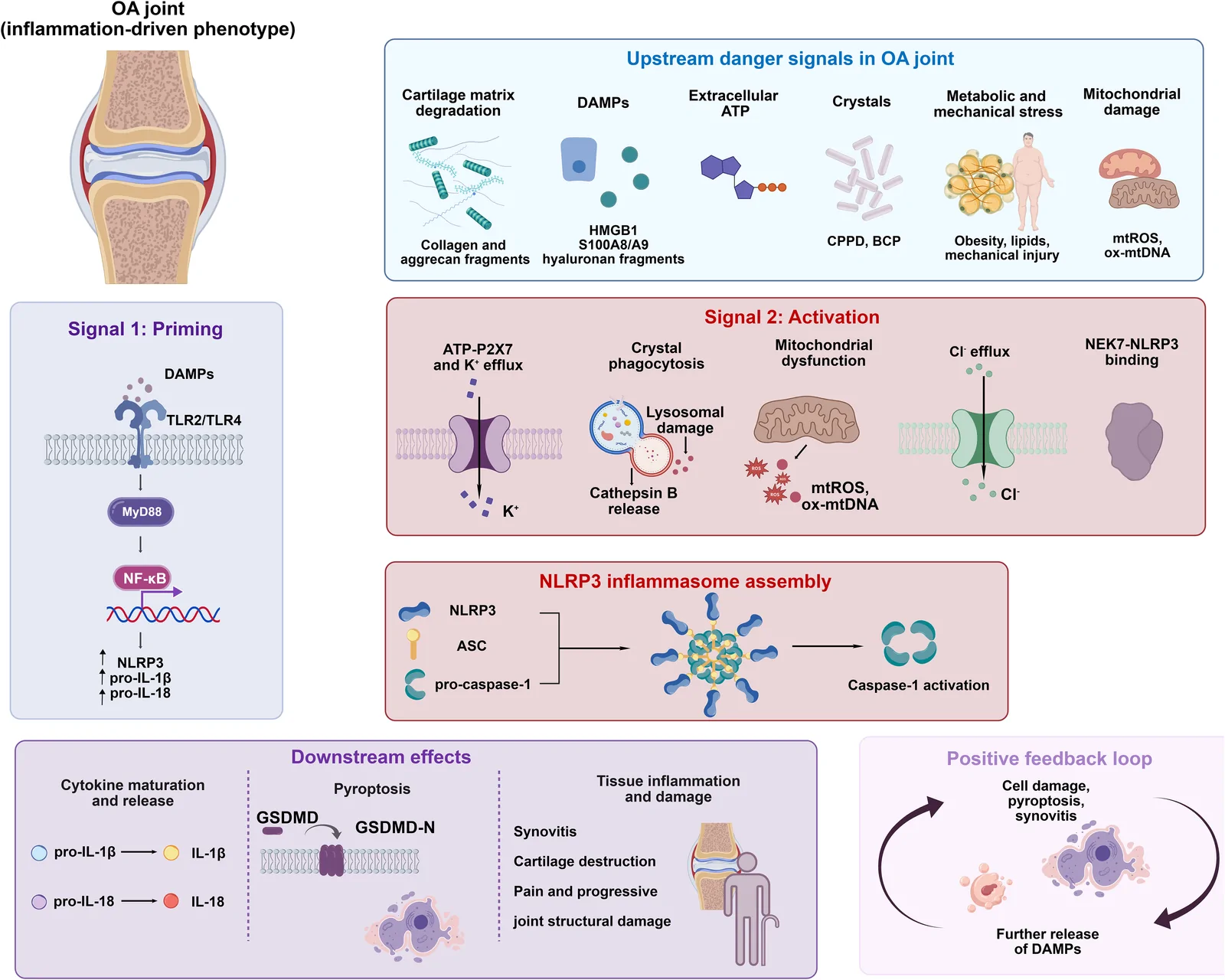
Canonical activation of the NLRP3 inflammasome and inflammatory amplification in inflammation-driven osteoarthritis. In the osteoarthritis (OA) joint microenvironment, cartilage matrix fragments, damage-associated molecular patterns (DAMPs), including high-mobility group box 1 (HMGB1), S100 calcium-binding proteins A8/A9 (S100A8/A9), and hyaluronan fragments, extracellular adenosine triphosphate (ATP), calcium-containing crystals including basic calcium phosphate (BCP) and calcium pyrophosphate dihydrate (CPPD), metabolic and mechanical stress, and mitochondrial damage may provide upstream danger signals. During priming (Signal 1), DAMP recognition by Toll-like receptor 2 (TLR2) and Toll-like receptor 4 (TLR4) activates myeloid differentiation primary response 88 (MyD88)–nuclear factor kappa B (NF-κB) signaling, increasing the expression of NOD-like receptor family pyrin domain-containing 3 (NLRP3), pro-interleukin-1β (pro-IL-1β), and pro-interleukin-18 (pro-IL-18). Activation stimuli (Signal 2), including ATP–purinergic receptor P2X7 (P2X7)-dependent K^+^ efflux, crystal phagocytosis-induced lysosomal damage and cathepsin B release, mitochondrial dysfunction with mitochondrial reactive oxygen species (mtROS) generation and oxidized mitochondrial DNA (ox-mtDNA) release, Cl^−^ efflux, and NIMA-related kinase 7 (NEK7)–NLRP3 interaction, promote assembly of NLRP3 with apoptosis-associated speck-like protein containing a caspase recruitment domain (ASC) and pro-caspase-1, leading to caspase-1 activation. Activated caspase-1 processes pro-IL-1β and pro-IL-18 into their mature forms and cleaves gasdermin D (GSDMD) to generate its pore-forming N-terminal fragment (GSDMD-N), resulting in cytokine release and pyroptosis. These processes may contribute to synovitis, cartilage destruction, pain, and progressive joint structural damage, while pyroptotic cell injury and subsequent DAMP release may further amplify local inflammation. Human OA samples support the presence of several upstream stimuli and NLRP3-related inflammatory mediators in affected joints; however, the detailed causal sequence of inflammasome activation and downstream effects depicted here is derived predominantly from mechanistic studies in cultured cells and animal models rather than being fully validated in human OA. Arrows indicate the direction of signaling events or feedback effects.

##### 6.1.2.2 Non-canonical pathway

The non-canonical pathway is primarily associated with intracellular Gram-negative bacterial infection, with lipopolysaccharide (LPS) serving as the central activating mediator. This process does not initially depend on caspase-1. Instead, LPS enters the cytosol through mechanisms such as bacterial uptake or experimental transfection (81, 82). Cytosolic LPS directly binds to the CARD domains of inflammatory caspases and induces their oligomerization and activation. Caspase-11 is the major mediator in mice, whereas caspase-4 and caspase-5 fulfill this role in humans (82). Activated caspase-4, caspase-5, and caspase-11 can directly cleave GSDMD, releasing the pore-forming GSDMD-N fragment. GSDMD-N oligomerizes at the plasma membrane, resulting in pore formation, ionic imbalance, leakage of intracellular contents, and pyroptotic cell death (81, 83). One study of macrophages exposed to Gram-negative bacterial infection suggested that the simultaneous recognition of bacterial mRNA and cytosolic LPS may promote the interaction between pro-caspase-11 and NLRP3, thereby enhancing non-canonical NLRP3 inflammasome activation. However, this mechanism requires further validation (84). OA joints are predominantly characterized by sterile inflammation, in which inflammatory responses are primarily driven by DAMPs released during cellular injury, extracellular matrix degradation, and tissue stress rather than by PAMPs such as LPS (85). However, under conditions such as obesity, metabolic syndrome, or impaired intestinal barrier function, gut-derived LPS may enter the systemic circulation and contribute to low-grade metabolic endotoxemia. Previous studies have detected LPS and LPS-binding protein in the serum and synovial fluid of patients with knee OA, with their levels being associated with intra-articular macrophage activation and OA severity (86, 87). Because cytosolic LPS can directly activate human caspase-4 and caspase-5, these specific metabolic conditions may provide a biological basis for the potential involvement of the non-canonical inflammasome pathway in OA. However, direct evidence demonstrating that circulating or gut-derived LPS enters the cytosol and induces caspase-4/5-dependent pyroptosis in OA is currently lacking, and this mechanism requires further investigation.

### 6.2 Evidence for aberrant NLRP3 inflammasome activation in OA

In recent years, the mechanisms through which the NLRP3 inflammasome contributes to OA have become increasingly clarified. Recent evidence from human samples, *in vitro* systems, and animal models has implicated the NLRP3 inflammasome in OA-associated inflammatory processes across multiple joint compartments. Extracellular mediators in the circulation and synovial fluid, together with cell-intrinsic activating signals in chondrocytes, fibroblast-like synoviocytes, and synovial macrophages, may promote NLRP3 inflammasome activation, while emerging evidence further suggests its involvement in subchondral bone remodeling. Collectively, these findings support a potential role for NLRP3-associated signaling within the whole-joint microenvironment of OA.

#### 6.2.1 Extracellular mediators

Multiple soluble mediators and crystal particles in the OA joint microenvironment may serve as second signals for NLRP3 inflammasome activation. Calcium-containing crystals appear to represent important extracellular activators (88). Hydroxyapatite has been detected in synovial fluid from patients with OA (89), whereas basic calcium phosphate (BCP) and calcium pyrophosphate dihydrate (CPPD) crystals have been identified in cartilage, synovial fluid, and meniscal tissue. These crystals can induce IL-1β production in a partially NLRP3-dependent manner and aggravate inflammatory joint pathology (90). Metabolic mediators may also participate in NLRP3 activation. In the context of obesity-related metabolic disease, type 2 diabetes, and metabolic syndrome, locally or systemically generated free fatty acids, ATP, and adenosine may trigger NLRP3-dependent inflammatory responses (91). Elevated levels of NLRP3, caspase-1, IL-1β, and IL-18 have been detected in synovial fluid from patients with temporomandibular joint OA (92). In an *in vitro* study using FLS isolated from knee OA joints, combined LPS and ATP stimulation significantly increased the mRNA expression of NLRP3, ASC, and CASP1, accompanied by increased soluble secretion of IL-1β and IL-18 (93). Notably, peripheral blood mononuclear cells (PBMCs) from patients with hand OA showed an overall reduction in IL-1β and NLRP3 transcript levels, without a significant increase in serum IL-1β. However, the NLRP3/β-actin ratio differed between non-erosive and erosive OA subtypes, suggesting that NLRP3-related inflammatory activity may vary according to OA subtype (94).

#### 6.2.2 Chondrocytes

Chondrocytes can activate the NLRP3 inflammasome through multiple mechanisms, including transcriptional regulation and oxidative stress. Studies have shown that C-terminal binding proteins (CtBP1 and CtBP2) are upregulated in chondrocytes from patients with OA. This may be related to reduced DNMT1 and DNMT3 expression and subsequent demethylation of the CTBP1 and CTBP2 genes. CtBP proteins can bind p300 and recruit AP-1 subunits to form a CtBP–p300–AP-1 transactivation complex, which directly binds the NLRP3 promoter and enhances NLRP3 transcription. This process subsequently promotes caspase-1 cleavage and IL-1β expression (95). Long non-coding RNA SNHG7 is considered a negative regulator of NLRP3 inflammasome activation and is downregulated in OA chondrocytes. Restoration of SNHG7 expression can suppress the miR-214-5p/PPARGC1B axis, thereby attenuating NLRP3 inflammasome activation, chondrocyte apoptosis, and inflammatory responses (96). In contrast, miR-30b-5p is significantly upregulated in OA joint tissues and chondrocytes from destabilization of the medial meniscus models. By targeting and inhibiting SIRT1, miR-30b-5p disrupts the SIRT1/FoxO3 axis, promotes chondrocyte apoptosis, and enhances NLRP3 inflammasome activation, thereby exacerbating OA progression (97).

Oxidative stress also contributes to NLRP3 activation in chondrocytes. Advanced glycation end products can reduce nuclear factor erythroid 2-related factor 2 (NRF2) expression, thereby relieving its inhibitory effects on NLRP3 and ASC transcription and promoting the secretion of IL-1β and IL-18 (98). Ubiquitin-specific protease 7 (USP7) promotes ROS production by reducing the ubiquitination-dependent degradation of NADPH oxidase 4 (NOX4). This process enhances NLRP3 and caspase-1 activation, GSDMD-mediated pyroptosis, and the expression of MMP-1 and MMP-13. Increased USP7 and NOX4 expression in cartilage from patients with OA further supports the potential relevance of this axis in joint pathology (99). IL-1β stimulation may additionally reduce miR-223-3p expression in chondrocytes, thereby further amplifying inflammatory responses (100). *In vitro*, MCC950 inhibited IL-1β-induced upregulation of NLRP3 and caspase-1 in chondrocytes and attenuated cartilage degeneration in murine OA models, supporting the involvement of NLRP3 in chondrocyte responses to inflammatory stimuli (101). P2X7 receptor activation can upregulate NLRP3 expression through NF-κB signaling, thereby promoting chondrocyte pyroptosis and cartilage matrix degradation. Pharmacological antagonism of P2X7 attenuates these pathological changes (102). In addition, activation of the liver kinase B1/AMP-activated protein kinase pathway can suppress NLRP3 inflammasome responses and reduce chondrocyte pyroptosis, further supporting the involvement of NLRP3 in chondrocyte inflammatory death and matrix destruction (103).

#### 6.2.3 Fibroblast-like synoviocytes

FLS are a major stromal cell population in the synovium and play an important role in OA-associated inflammation. IL-1β, a key downstream effector of the NLRP3 inflammasome, not only participates in inflammatory amplification but also promotes FLS activation. Under LPS stimulation, NLRP3-deficient FLS produce significantly lower levels of IL-1β and IL-18, supporting the presence of a functional NLRP3-dependent inflammatory signaling pathway in these cells (104). The hypoxia-inducible factor-1α-mediated hypoxic signaling axis is also involved in NLRP3 inflammasome regulation. Increased hypoxic stress can activate HIF-1α, which has been associated with enhanced NLRP3 inflammasome activation, exacerbated synovial inflammation, and tissue fibrosis (105, 106). In an *in vitro* experimental system using cultured FLS derived from human knee OA tissue, combined LPS and ATP stimulation significantly increased NLRP3, ASC, and CASP1 mRNA expression, accompanied by elevated soluble IL-1β and IL-18 levels (93). In RA, the activity of the NLRP3 inflammasome in FLS appears to be relatively limited. Available evidence suggests that NLRP3 inflammasome expression levels in FLS from patients with RA remain largely unchanged, and its activation does not induce IL-1β secretion, exhibiting distinct characteristics compared with OA-FLS (107). Furthermore, pyroptosis-derived danger signals released during OA progression may reactivate the NLRP3 inflammasome in FLS, thereby establishing a positive feedback loop between cartilage and synovium that continuously amplifies inflammatory responses within synovial tissues (108, 109).

#### 6.2.4 Synovial macrophages

Recent studies suggest that synovial macrophages are important effector cells for NLRP3 inflammasome activation within the OA synovial microenvironment (110, 111). LPS and ATP stimulation can induce mitochondrial morphological damage, loss of mitochondrial membrane potential, impaired activity of respiratory-chain complexes I–IV, and excessive ROS accumulation in macrophages. These changes represent important upstream signals for NLRP3 inflammasome activation. In preclinical experimental models, treatment with the hydrogen sulfide donor GYY4137 improves mitochondrial function and markedly reduces NLRP3, active caspase-1, and GSDMD-N expression in synovial macrophages. This intervention suppresses pyroptosis and alleviates OA-associated synovitis and joint injury (112). Macrophages in synovial tissue from patients with OA can also undergo GSDMD-dependent pyroptosis accompanied by increased ROS production. This process is associated with aberrant mTORC1 activation and increased MMP-9 expression, suggesting that macrophage pyroptosis may contribute to synovial inflammation and tissue remodeling (113). Taken together, current evidence supports a model in which mitochondrial injury and ROS accumulation sustain NLRP3 inflammasome activation in synovial macrophages, leading to pyroptosis and pro-inflammatory cytokine release, thereby promoting local inflammation and joint tissue destruction.

#### 6.2.5 Subchondral bone

In addition to articular cartilage and synovium, subchondral bone remodeling represents an important component of the whole-joint pathological process in OA. Emerging evidence suggests that subchondral bone contributes to OA progression through bone resorption, the release of inflammatory mediators, and intercellular signaling, with abnormal osteoclast activation and the RANKL/RANK/OPG signaling axis serving as key regulators of subchondral bone remodeling (114). Several studies have suggested that the NLRP3 inflammasome may participate in the regulation of osteoclast function, and that its activation can enhance osteoclast activity by promoting inflammatory responses, aggravating oxidative stress, and strengthening bone resorption-related signaling. Conversely, RANKL-induced ROS accumulation and the local inflammatory microenvironment may further facilitate NLRP3 inflammasome activation (115). However, current evidence regarding the specific role of NLRP3 in OA subchondral bone is derived mainly from experimental studies, and its functional significance in human osteoclasts and osteocytes, as well as its clinical relevance, remains insufficiently validated. Future studies integrating analyses of cartilage, synovium, and subchondral bone are therefore warranted to further clarify the role of NLRP3 within the whole-joint microenvironment of OA (**Figure 2**).

**Figure 2:**
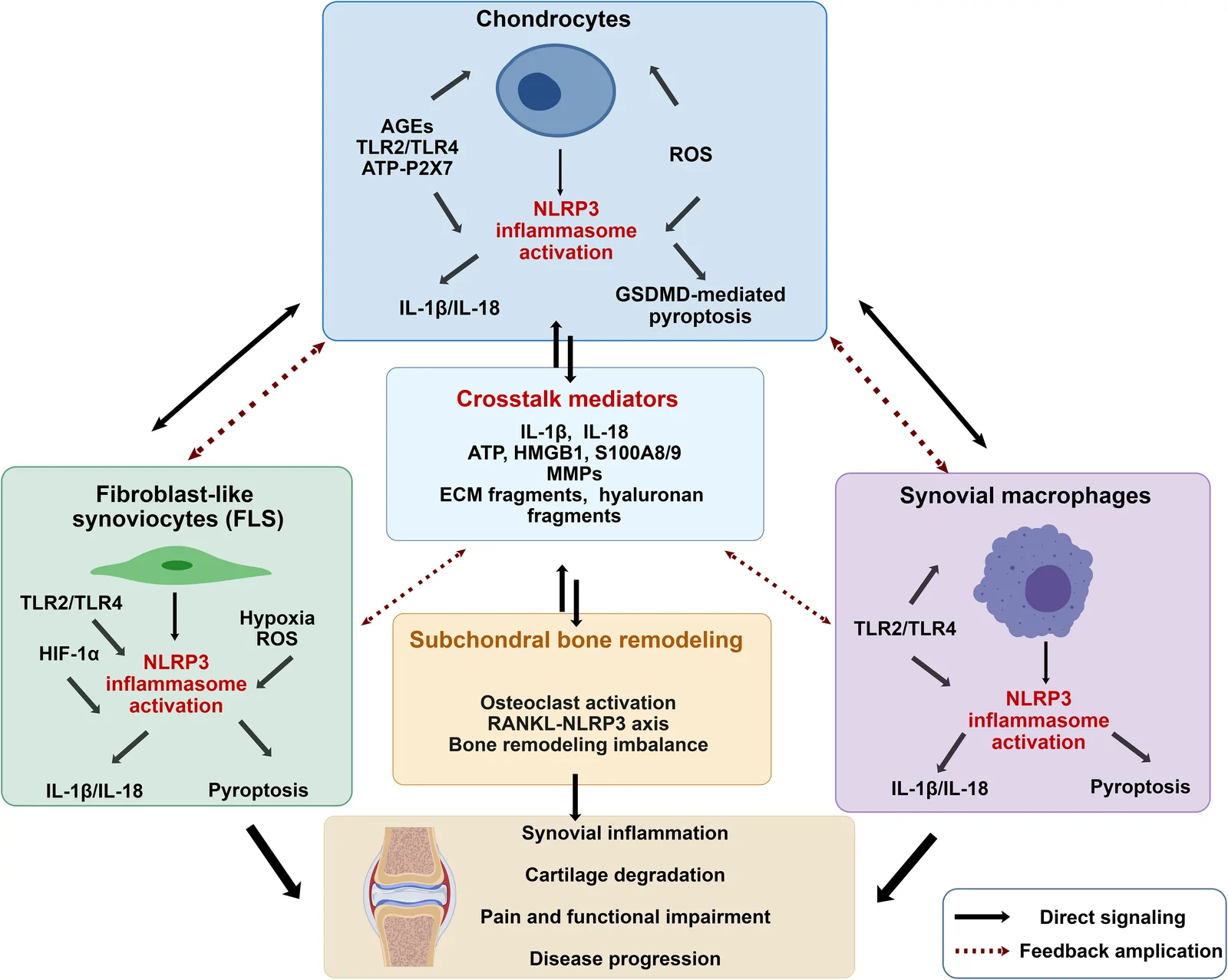
NLRP3 inflammasome activation and intercellular crosstalk in the OA joint microenvironment. In chondrocytes, advanced glycation end products (AGEs), Toll-like receptor 2/Toll-like receptor 4 (TLR2/TLR4) signaling, adenosine triphosphate (ATP)-purinergic receptor P2X7 (P2X7) signaling, and reactive oxygen species (ROS) contribute to NOD-like receptor family pyrin domain-containing 3 (NLRP3) inflammasome activation, resulting in interleukin-1β (IL-1β) and interleukin-18 (IL-18) release and gasdermin D (GSDMD)-mediated pyroptosis. In fibroblast-like synoviocytes (FLS), TLR2/TLR4 signaling, hypoxia-inducible factor 1 alpha (HIF-1α)-associated hypoxic stress, and ROS promote NLRP3 activation, inflammatory cytokine release, and pyroptosis. In synovial macrophages, innate immune stimuli and mitochondrial oxidative stress contribute to NLRP3 activation, IL-1β/IL-18 release, and pyroptosis. Inflammatory mediators released from these cells, including IL-1β, IL-18, ATP, high-mobility group box 1 (HMGB1), S100 calcium-binding proteins A8/A9 (S100A8/A9), matrix metalloproteinases (MMPs), extracellular matrix (ECM) fragments, and hyaluronan fragments, mediate intercellular communication and amplify joint inflammation, cartilage degradation, pain, functional impairment, and OA progression. In subchondral bone, NLRP3-associated inflammatory signaling may contribute to osteoclast activation and abnormal bone remodeling, although current evidence is mainly derived from experimental models. Solid black arrows indicate direct signaling or downstream effects, whereas red dotted arrows indicate indirect feedback amplification mediated by inflammatory mediators and damage-associated signals. Human OA tissues and patient-derived cells support the presence of NLRP3-associated abnormalities in chondrocytes, FLS, and synovial macrophages; however, most causal relationships linking specific upstream stimuli, inflammasome activation, pyroptosis, and intercellular feedback have been established primarily in cultured cells, co-culture systems, and animal models.

### 6.3 Potential of NLRP3 inflammasome activation as a candidate research biomarker for inflammation-driven OA

Although the contribution of inflammation to OA initiation and progression has been increasingly recognized, specific diagnostic markers for inflammation-driven OA remain unavailable (116).

Current approaches to identifying inflammation-driven OA largely rely on imaging findings and the expression of serum inflammatory cytokines (7). Advanced imaging techniques, including musculoskeletal US and MRI, can detect synovitis in OA joints and have contributed substantially to the understanding of inflammatory processes in OA (117, 118). Imaging features such as synovial thickening, joint effusion, and synovial blood flow are widely used to assess local inflammatory activity. Their semiquantitative measures are significantly associated with pain severity and disease progression (119). A 4-year follow-up analysis of the Osteoarthritis Initiative cohort demonstrated that persistent synovitis was associated with structural damage in knee OA (120). In addition, [18F] NaF PET-MRI has provided direct evidence linking regions of active bone metabolism with adjacent synovitis in knee OA (121). Multiple studies have also confirmed the involvement of inflammatory mediators in peripheral blood and synovial fluid during OA progression (122–124). Comparative analyses of synovial fluid from patients with OA, RA, and normal knees showed that IL-4, IL-6, IL-8, and IL-15 concentrations were substantially higher in OA synovial fluid than in matched serum samples, indicating marked local inflammatory activation within the joint (125). Cytokine and matrix-degrading enzyme profiles in OA synovial fluid further support the existence of an intra-articular pro-inflammatory microenvironment and suggest that synovial fluid inflammatory markers may contribute to patient subgroup identification and inflammatory phenotype stratification. Cytokines and adipokines have also been proposed as potential OA biomarkers. These molecules may promote cartilage degeneration and are associated with pain-related measures, including higher Western Ontario and McMaster Universities Osteoarthritis (WOMAC) index scores (126, 127).

However, accumulated evidence regarding pro-inflammatory mediator expression and its association with clinical and imaging features remains insufficient to independently define inflammation-driven OA. Imaging primarily reflects tissue and structural changes resulting from local inflammatory activity. Although it can indicate the presence of inflammation, it cannot reliably distinguish inflammation driven by intrinsic OA mechanisms from synovitis occurring secondarily to mechanical stress or structural damage. In addition, synovitis severity may be influenced by age, obesity, genetic background, and other individual factors. Therefore, defining an inflammation-driven OA phenotype solely on the basis of imaging changes has inherent limitations (8). Whether limitations in current inflammation-based stratification contribute to the variable responses observed with anti-inflammatory therapies remains to be established. Several biochemical markers, including C-terminal cross-linked telopeptide of type II collagen, cartilage oligomeric matrix protein, and MMP-3, have been proposed to assist in predicting OA progression and evaluating responses to emerging therapies (128). Nevertheless, because of the heterogeneity of OA pathogenesis and the complexity of its clinical manifestations, no clinically validated biomarker is currently available for OA diagnosis, disease monitoring, or prognosis. Welhaven et al. noted that no molecular marker has yet been incorporated into routine OA diagnostic or monitoring frameworks (129). Moreover, individual biomarkers may not adequately reflect a specific pathological endotype (6), and the reproducibility of many candidate biomarkers across independent cohorts remains inconsistent. For example, periostin (POSTN) has been implicated in OA pathogenesis, and chondrocyte-derived POSTN may have value in disease diagnosis and severity stratification. However, serum POSTN levels were not significantly associated with WOMAC index scores or Kellgren-Lawrence (KL) grades. Further analyses suggested that serum POSTN was not a significant predictor of OA outcomes (128).

As an inflammatory signaling hub linking danger-signal recognition, innate immune activation, and cytokine maturation and release, the NLRP3 inflammasome may warrant investigation as a candidate research biomarker for inflammation-driven OA. NLRP3 activation occurs at an upstream node of the inflammatory signaling cascade. In addition to regulating the release of mature cytokines such as IL-1β and IL-18, it mediates GSDMD-dependent pyroptosis. Therefore, compared with downstream cytokines alone, NLRP3 inflammasome activation may more closely reflect inflammatory initiation mechanisms rather than merely the consequences of established inflammation (49). Moreover, the NLRP3 inflammasome can integrate multiple danger signals within the OA joint microenvironment, including crystal deposition, ATP release, K^+^ efflux, mitochondrial ROS accumulation, HMGB1 release, and extracellular matrix (ECM) degradation products (58, 130). Its activation status may therefore capture the convergence of multiple inflammatory stimuli and the overall level of innate immune activation more effectively than a single cytokine measurement. This feature provides a potential rationale for investigating whether NLRP3-related activity can contribute to the research stratification of OA patients with persistent innate immune activation.

NLRP3-based biomarkers still face multiple practical challenges that may limit their clinical translation. Regarding sample sources, synovial fluid can directly reflect the local inflammatory microenvironment of the joint and allows the detection of NLRP3, caspase-1, IL-1β, and IL-18. However, its acquisition requires joint aspiration and is influenced by several factors, including synovial fluid volume, viscosity, dilution effects, and blood contamination, limiting its feasibility for large-scale screening and longitudinal monitoring (131, 132). Although peripheral blood is more accessible and suitable for repeated sampling, circulating levels of NLRP3-related molecules may be affected by systemic inflammatory status, metabolic factors, and contributions from extra-articular tissues. It remains unclear whether circulating NLRP3 and its downstream effectors can reliably and quantitatively reflect local inflammasome activation within OA joints. Furthermore, NLRP3 transcriptional levels or total protein expression do not necessarily indicate functional inflammasome activation. Therefore, future candidate biomarker panels may require the combined assessment of functional indicators reflecting inflammasome activity, including ASC speck formation, activated caspase-1, the GSDMD-N fragment, and mature IL-1β/IL-18. These functional readouts can be evaluated using different approaches depending on sample types, including western blotting, immunofluorescence, immunohistochemistry, ELISA, or flow cytometry. However, standardized protocols for detection methods, sample processing procedures, and threshold definitions have not yet been established.

Current evidence supporting NLRP3 activation as a biomarker for OA is primarily derived from small-scale cross-sectional studies or exploratory investigations. Large-scale, multicenter, prospective cohort studies for independent validation remain lacking, and paired analyses among peripheral blood, synovial fluid, and joint tissues from the same individuals are also limited. Therefore, future studies should establish standardized sampling strategies and longitudinal study designs to further determine the consistency of NLRP3 activation-related indicators across different biological sources and to clarify their associations with local imaging-based inflammatory features, histopathological alterations, disease progression, and therapeutic responses.

Collectively, current NLRP3 research in OA has focused predominantly on its role in inflammatory pathogenesis. Its clinical translational value as a marker of inflammation-driven OA has not been sufficiently validated (56). Whether NLRP3 inflammasome activation can be used to identify OA patients with persistent inflammatory activity, predict structural or symptomatic progression, and select individuals most likely to benefit from anti-inflammatory or NLRP3-targeted therapies remains unclear, warranting systematic mechanistic studies and well-designed prospective clinical investigations.

## 7 The NLRP3 inflammasome: a potential therapeutic target in inflammation-driven OA

### 7.1 Advances in therapeutic strategies targeting the NLRP3 inflammasome

Current OA management remains primarily focused on symptomatic pain relief, and DMOADs that can effectively delay or prevent disease progression are still lacking (3). As preclinical evidence has increasingly implicated the NLRP3 inflammasome in inflammatory amplification, pyroptosis, and joint tissue damage, targeting this pathway has become an active area of OA therapeutic research (12). Existing approaches mainly focus on inhibiting upstream NLRP3-activating signals, interfering with inflammasome activation and assembly, or suppressing downstream pyroptotic effects.

Accumulating evidence indicates that DAMPs, including calcium-containing crystals and elevated extracellular ATP, can drive and regulate NLRP3 inflammasome activation (102). Phosphocitrate (PC), an inhibitor of calcium-containing crystal formation, has shown therapeutic activity in improving OA-related pathological features in several studies (133). Its structural analogue, Carolinas Molecule-01 (CM-01), can suppress calcium crystal-associated inflammatory processes. In Hartley guinea pig models, CM-01 treatment attenuated cartilage degeneration, osteophyte formation, and meniscal calcification (134). In addition, the ATP-activated purinergic receptor P2X7 can initiate NLRP1- and NLRP3-dependent inflammatory responses in OA (102, 135, 136). The P2X7 receptor antagonist AZD9056 demonstrated therapeutic effects in monosodium iodoacetate-induced (MIA) rat OA models, including reduced P2X7 expression, modulation of NF-κB signaling, and suppression of pro-inflammatory mediator production (137). These findings suggest that blocking upstream inflammasome-activating signals may alleviate local inflammatory responses in OA.

Oxidative stress is considered an important link connecting mechanical injury, metabolic dysregulation, and inflammatory amplification. In particular, ROS accumulation can promote NLRP3 inflammasome assembly and activation. The USP7–NOX4–ROS–NLRP3 signaling axis has been implicated in OA-associated inflammatory responses, and inhibition of different components of this axis may exert therapeutic effects in OA chondrocytes (99). The ROS inhibitor Apocynin can reduce NLRP3 activation and pyroptosis in chondrocytes, while decreasing the expression of MMP-1 and MMP-3 (99). Nrf2, as an important antioxidant transcription factor, can negatively regulate NLRP3 inflammasome activation (138). Several natural bioactive compounds, including Quercetin (139), Icariin (140), and Sinomenine (100), have been reported to activate NRF2 signaling or attenuate oxidative stress, thereby reducing the expression of NLRP3, ASC, and caspase-1 and alleviating cartilage damage and inflammatory responses. However, most of these studies remain limited to cell-based and animal experiments. Nevertheless, they provide potential therapeutic approaches for regulating NLRP3 inflammasome activation.

Compared with indirect regulatory strategies, direct inhibition of the NLRP3 inflammasome is considered to offer greater target specificity. MCC950 is a widely investigated selective NLRP3 inhibitor that suppresses inflammasome assembly by binding to a specific site within the NLRP3 NACHT domain (141). In IL-1β-stimulated chondrocytes, MCC950 suppresses the induction of NLRP3 and caspase-1, reduces the expression of inflammatory mediators and matrix-degrading enzymes, and attenuates oxidative stress-induced injury. In a murine OA model, intra-articular administration of MCC950 alleviates cartilage degeneration (101, 142). These findings provide preclinical evidence supporting direct NLRP3 inhibition as a potential therapeutic strategy in OA, but do not establish clinical efficacy.

Pyroptosis is an inflammatory form of programmed cell death induced downstream of NLRP3 inflammasome activation (143). By promoting the release of IL-1β, IL-18, and other inflammatory mediators, pyroptosis can accelerate ECM degradation and may represent an important therapeutic target in OA. As the key executioner of pyroptosis, GSDMD is upregulated in OA synovial and cartilage tissues. Several agents, including disulfiram and glycyrrhizin, have been reported to reduce inflammatory cytokine release and pyroptosis by inhibiting GSDMD activation or pore formation (144–146) (**Table 1**).

**Table 1:** Representative pharmacological agents targeting the NLRP3 inflammasome axis in osteoarthritis.

Therapeutic target	Representative compound	Proposed mechanism	Experimental model	References
Calcium crystal-associated DAMPs	Phosphocitrate (PC)	Inhibits calcium crystal formation and associated inflammation	Hartley guinea pig OA	(133)
	CM-01 (PC analogue)	Inhibits calcium crystal- associated inflammation	Hartley guinea pig OA	(134)
P2X7R	AZD9056	Antagonizes P2X7R and suppresses NF-κB-associated inflammatory signaling	MIA rat OA	(137)
ROS	Apocynin	Suppresses ROS production and reduces NLRP3 inflammasome activation and pyroptosis	Rat primary chondrocytes (H_2_O_2_)	(99)
NLRP3-related pyroptosis	Quercetin	Reduces oxidative stress and suppresses NLRP3-mediated pyroptosis	C28/I2 chondrocytes (LPS); ACLT/PMM rat OA	(139)
	Icariin	Suppresses NLRP3/caspase-1-mediated pyroptotic signaling	Rat chondrocytes (LPS); MIA rat OA	(140)
miR-223-3p/NLRP3	Sinomenine	Upregulates miR-223-3p and suppresses NLRP3 inflammasome signaling	Chondrocytes (IL-1β); ACLT mouse OA	(100)
NLRP3 inflammasome	MCC950	Selectively inhibits NLRP3 inflammasome activation	Mouse chondrocytes (IL-1β); DMM mouse OA	(101)
GSDMD-mediated pyroptosis	Disulfiram	Inhibits GSDMD-mediated pyroptotic signaling	C28/I2 chondrocytes (LPS/ATP)	(145)

ACLT, anterior cruciate ligament transection; MIA, monosodium iodoacetate; PMM, partial medial meniscectomy; DMM, destabilization of the medial meniscus.

Taken together, targeting the NLRP3 inflammasome and its downstream signaling pathways represents a promising therapeutic strategy for OA. Natural compounds and synthetic small-molecule inhibitors may attenuate joint inflammation and delay disease progression by suppressing NLRP3 inflammasome activation, highlighting their therapeutic potential (147). However, current evidence is derived predominantly from *in vitro* experiments and preclinical animal models. Whether these interventions can modify established OA pathology and provide meaningful clinical benefit requires further validation.

### 7.2 Prospects for NLRP3 inflammasome-informed precision therapy in inflammation-driven OA

Inflammation-driven OA may be characterized by persistent innate immune activation and a sustained inflammatory state. As a central node linking danger signal recognition to caspase-1 activation, IL-1β and IL-18 maturation and release, and pyroptosis, the activation status of the NLRP3 inflammasome may reflect the overall activity of local inflammatory cascades. Accordingly, NLRP3-related activity warrants further investigation as a candidate research marker for inflammation-driven OA. Previous clinical trials targeting IL-1 or TNF-α have failed to demonstrate significant therapeutic efficacy in OA populations. These findings may suggest that the limited efficacy of these approaches does not necessarily reflect inappropriate target selection, but may instead indicate the current lack of precise identification of inflammation-driven OA subgroups (35, 116, 148). However, it should be emphasized that attributing the poor therapeutic response observed in unselected OA cohorts to insufficient subtype stratification remains a hypothesis associated with NLRP3 activation status. This concept currently lacks direct support from human studies and should not be considered an established conclusion. Current approaches for identifying inflammation-driven OA remain limited. In this review, we propose that aberrant NLRP3 inflammasome activation during chronic, persistent low-grade inflammation may represent a potential entry point for OA stratification and precision treatment.

Preclinical evidence supports the NLRP3 inflammasome as a potential therapeutic target in OA; however, its clinical utility remains to be established, and whether targeting this pathway can provide clinical benefit specifically in inflammation-driven OA remains unknown (56). Importantly, it should be noted that direct targeting of the NLRP3 inflammasome may also entail potential risks. Studies have shown that Nlrp3-deficient mice exhibit enhanced autophagy under both basal and stress conditions, suggesting that NLRP3 may participate in autophagy regulation and other homeostatic functions under physiological conditions (149, 150). Prolonged and complete inhibition of NLRP3 activity may impair innate immune responses to stimuli such as DAMPs and potentially induce compensatory activation of other innate immune-related pathways (151). A previous study demonstrated that pharmacological inhibition of NLRP3 by MCC950 in a hypoxia-induced microglial model did not significantly reduce IL-1β production, but instead was accompanied by increased expression of NLRC4 and AIM2 proteins, suggesting potential compensatory regulation among different inflammasomes (152, 153). Furthermore, in viral infection models, impaired NLRP3 inflammasome activation and pyroptosis have been associated with enhanced caspase-8/RIPK3-dependent inflammatory cell death, indicating that inflammatory responses may be sustained through alternative cell death programs (153). Recent studies have suggested that other inflammasomes, such as AIM2, may also participate in inflammatory responses within arthritic conditions. In addition, cGAS-STING, an independent cytosolic DNA-sensing pathway, has been shown to contribute to inflammatory responses and mechanical hyperalgesia in experimental mouse OA models (154, 155). However, direct evidence demonstrating that pharmacological inhibition of NLRP3 induces compensatory activation of AIM2, NLRC4, cGAS-STING, or caspase-8/RIPK3 pathways in OA remains lacking. It remains unclear whether these pathways represent parallel inflammatory mechanisms coexisting with NLRP3 in OA or whether they can be actively induced as compensatory responses following NLRP3 inhibition. Such potential pathway compensation may have important implications for the efficacy and safety of NLRP3-targeted therapies in OA. On the one hand, persistent activation of alternative inflammatory pathways may reduce the durability of therapeutic effects achieved by selective NLRP3 inhibition. On the other hand, broad suppression of multiple inflammatory pathways to prevent potential compensatory activation may expand the extent of immune modulation and increase the risk of infection or disruption of immune homeostasis during long-term treatment. Therefore, future OA therapeutic development should further elucidate the dynamic remodeling of inflammatory networks following NLRP3 inhibition and systematically evaluate whether localized, partial, or temporally controlled modulation of NLRP3 activity may represent a more feasible strategy than prolonged and complete blockade.

Selective inhibition represents another important issue that requires further investigation. Biological agents, including IL-1β inhibitors, can partially alleviate inflammatory responses; however, they do not directly block NLRP3 inflammasome assembly and therefore cannot completely prevent sustained IL-1β production (156, 157). Canakinumab is a monoclonal antibody against IL-1β that exerts anti-inflammatory effects primarily by binding and neutralizing mature extracellular IL-1β, thereby preventing its interaction with the IL-1 receptor (157). However, its anti-inflammatory activity is limited by its inability to inhibit sustained caspase-1 activation, pro-IL-1β cleavage, and GSDMD-mediated pyroptosis downstream of NLRP3 inflammasome activation (63). Canakinumab has been approved for several inflammatory disorders, including cryopyrin-associated periodic syndromes (CAPS) and tumor necrosis factor receptor-associated periodic syndrome (TRAPS) (158, 159). In the phase III CANTOS trial, canakinumab administered at 150 mg every 3 months reduced the risk of recurrent cardiovascular events but was associated with increased rates of thrombocytopenia and neutropenia, suggesting that long-term systemic IL-1β blockade may increase the risk of infection (160). In addition, differences in NLRP3-related genetic polymorphisms across populations of distinct genetic ancestries suggest that future NLRP3 gene-targeted strategies may need to be adapted to specific populations (161, 162). Overall, NLRP3-targeted therapeutic strategies remain at an exploratory stage. Their safety and efficacy require validation in large-scale, high-quality studies to provide a more robust basis for clinical translation and application.

## 8 Discussion and future perspectives

Research on inflammatory mechanisms in OA has advanced substantially in recent years. However, several challenges and unresolved questions remain in this field. First, inflammation-driven OA is currently defined in most studies based on imaging evidence of synovitis, inflammatory mediator levels, or histological characteristics, while a unified clinical diagnostic criterion has not yet been established. Moreover, these indicators mainly reflect downstream consequences of inflammation rather than the underlying driving mechanisms. The lack of standardized criteria for defining inflammation-driven OA across different studies reduces comparability among cohorts and limits the development of phenotype-based therapeutic strategies. Although the TLR–NF-κB axis, DAMP recognition, and mechanisms of NLRP3 inflammasome activation have been increasingly clarified, the temporal and spatial integration of these signals, as well as the dynamic networks formed among chondrocytes, synovial macrophages, and fibroblast-like synoviocytes, remain insufficiently understood.

In the therapeutic field, most strategies targeting the NLRP3 inflammasome remain at the stage of laboratory investigation and preclinical animal studies, and substantial barriers remain between basic research and clinical application. Although numerous small-molecule inhibitors targeting different components of the NLRP3 inflammasome pathway have been developed, their pharmacokinetic properties, *in vivo* efficacy, and safety require further validation. Agents that demonstrate efficacy in animal models may produce different responses in human clinical studies, including adverse effects, immunogenicity, and other safety concerns. More importantly, the lack of clinical trial designs specifically focused on inflammation-driven OA may dilute potential therapeutic effects because of patient heterogeneity and obscure the true benefit of targeted interventions. This limitation has substantially constrained the current understanding of precision stratification and targeted treatment strategies for OA.

Future studies should first focus on establishing reproducible criteria for defining inflammation-driven OA. Multidimensional classification criteria integrating synovial pathological features, imaging findings, and molecular indicators are needed. Such frameworks should also clarify the relative contribution of NLRP3 inflammasome activation to inflammatory OA subtypes. In parallel, exploratory biomarker panels capable of reflecting NLRP3 activation status should be developed and systematically validated. Multi-omics technologies, including transcriptomics, proteomics, and metabolomics, may facilitate the comprehensive identification of molecular features associated with NLRP3 activation, caspase-1 activity, and GSDMD-mediated pyroptosis. The application of single-cell and spatial transcriptomic technologies may further clarify interactions among distinct cell populations within the OA joint microenvironment. In particular, it will be important to determine whether an NLRP3-centered inflammatory amplification circuit exists among synovial macrophages, chondrocytes, and FLS, and whether DAMPs derived from different cellular sources exert distinct signal-driving effects. In addition, the potential involvement of other inflammasomes in OA has received increasing attention in recent years. The AIM2 inflammasome recognizes aberrantly accumulated cytosolic double-stranded DNA and mediates downstream inflammatory responses (155). Studies using cellular senescence models have shown that AIM2 downregulation can attenuate senescence-associated phenotypes and pyroptosis-related changes, suggesting a potential role in age-related inflammatory processes (163). However, direct evidence supporting the involvement of AIM2 in OA remains limited, particularly with respect to validation of a causal relationship with cellular senescence in human joint tissues. Therefore, when evaluating NLRP3-targeted interventions, other inflammatory pathways and their potential interactions with NLRP3 should also be taken into consideration. At the clinical translational level, prospective studies based on distinct OA subtypes are urgently needed. Future clinical trials should avoid treating all patients with OA as a uniform population and should instead evaluate prospectively defined inflammatory features, such as persistent synovitis, together with exploratory NLRP3-related readouts or pyroptosis-associated signals. This approach may improve the ability to identify patients most likely to benefit from anti-inflammatory therapies. Prospective validation will therefore be required to determine whether NLRP3-related activity can provide incremental value within a multidimensional framework for OA stratification and treatment selection.

## 9 Conclusion

OA is characterized by marked clinical heterogeneity, and broadly applied anti-inflammatory therapies without phenotype-informed patient selection may not produce consistent clinical benefit. Therefore, identifying patients with persistent inflammatory features may be important for determining whether phenotype-informed patient selection can improve responses to anti-inflammatory treatment. Current evidence suggests that inflammation-driven OA is not merely a secondary consequence of mechanical injury or degenerative changes. Instead, it may involve multiple mechanisms, including sustained release of DAMPs, aberrant innate immune activation, and local inflammatory amplification. As a central hub linking danger signal recognition, caspase-1 activation, maturation and release of IL-1β and IL-18, and GSDMD-mediated pyroptosis, the NLRP3 inflammasome may participate in positive feedback loops between inflammation and tissue damage in chondrocytes, fibroblast-like synoviocytes, and synovial macrophages. Compared with individual downstream inflammatory cytokines, the activation status of the NLRP3 inflammasome may provide mechanistic information on innate immune activation in inflammation-driven OA. It therefore warrants further investigation as a candidate research biomarker and potential therapeutic target. However, its utility for clinical stratification has not been established, and the clinical efficacy and long-term safety of NLRP3-targeted therapies in OA remain to be determined.
